# Insurance Scraps of Paper

**Published:** 1923-04

**Authors:** R. W. Harris


### INSURANCE SCRAPS OF PAPER.

**By R. W. HARRIS.**

THERE are some millions of little pieces of paper handed out every year by doctors to their insured patients for them to transmit to their Societies. Every doctor who issues one of these little pieces of paper makes the specific statement that he has examined the patient on the date entered on the paper and the further express statement that the patient is, in the doctor's opinion, incapable of work. This piece of paper, this certificate, is the connecting link between medical benefit—the insurance benefit which is given in kind—and the two principal cash benefits, sickness and disablement benefits. It brings the doctors and the Societies into close relationship, though not necessarily into direct communication. It brings them, unfortunately, at times into conflict.

### A Valuable Document.

The certificate is a valuable document, and it is of great importance that every doctor should so regard it. The total amount paid away every year in sickness and disablement benefits is over £11,000,000. It stands to reason that, as the incapacity from work which entitles the insured person to receive the cash payment must be incapacity resulting from "some specific disease or bodily or mental disablement," this very considerable sum must be paid mainly on the strength of the medical certificates given by the panel doctors, notwithstanding the fact that the medical certificate does not confer upon the insured person any title to receive payment of benefit and that the Society may reject the evidence in favour of something which in their view is of more value, or send the case to the Medical Referee. If one of the Society representatives at the Guildhall Conference is correctly reported as having said that Society officials would be glad to do away with certificates altogether, but that they were faced with the requirements of the Government departments, he was unburdening himself of a particularly foolish observation. If doctors were encouraged to think that certificates were a mere matter of form it would be a sad thing for the funds of the Societies and for the good name of National Health Insurance. There is already evidence that too many doctors, owing to their very familiarity with the forms of certificate, get into a habit of mind with regard to them not far removed from the contempt which familiarity is said to breed. The with holding of a substantial sum from the doctor's pay comes sometimes as a sharp reminder of the importance of the statements to which the doctor has appended his signature.

### What is a Technical Irregularity ?

Every time that a doctor gives an incorrect certificate he explains when the day of reckoning comes that he has committed merely a technical irregularity. Now, one can sympathise with a busy doctor who is confronted with a request from an agitated applicant that he shall give a certificate in respect of a patient known to him as a chronic invalid, or as being in an Institution, coupled perhaps with a statement that the Society's agent "says that it will be all right," and that if the doctor does not sign the form the unfortunate patient will be deprived of his sick-pay. But the sympathy should only fairly extend to a doctor new to the work to whom such a request was made for the first time. At this date it certainly ought not to be possible for any intelligent man to go on thinking that the signing with his own hand of the statement, "I have examined you on the undermentioned date," when he has not in fact seen the patient on that date, can be regarded as a technical irregularity. I feel sure that Dr. Dain, when he pleaded at the Guildhall for the doing away with penalties for technical offences, must have had something else in mind. No Society representative could have wished his point of view better put than it was by Dr. Dain when he said that the certificate must be a document above suspicion and absolutely trustworthy. I could wish, at the same time, that a much wider audience of Society officials could have listened to Dr. Dain's plea for a better appreciation on their part of the difficult circumstances in which doctors often have to give certificates.

### A Question of Psychology.

There will, I am sure, be entire agreement with the proposition that nothing which weakens the force of the certificate in the mind of the doctor who is called upon to give it, or which fosters the idea that it is a mere formality, should be tolerated. And yet the statement of this self-evident proposition brings me hard up against a question of psychology which is really rather disturbing. What must be the effect on the minds of the doctors as a whole to find themselves day by day required—as they are required—to certify as "incapable of work" persons who are not, in fact, incapable of work ? I am aware that various shades of meaning can be found in the dictionaries as attaching to the word "incapable," but it is, I think, unquestionable that in ordinary parlance the expression "incapable of work" can only describe a condition which is not that of the great majority of persons who are given, and quite properly given, certificates for periods when they are not confined to bed in the acute stages of a severe illness. If the terms of the Statute were to be amended so that the doctor would in future have to certify that the patient is "rendered unfit for work by" a specified disease or disablement, the funds of the Societies, in my humble judgment, would not suffer. I am well aware that, in a psychological question of this kind, with such important bearings, there is room for considerable difference of opinion. But psychological considerations are too often not given their fair weight in discussing practical matters. If a general review of the certification system takes place, a frank examination of the question of the continuance of the phrase "incapable of work" is worth the while of all who agree that a certificate should be "absolutely trustworthy."

